# The President’s Case

**Published:** 1901-10

**Authors:** 


### TEXAS MEDICAL JOURNAL.

AUSTIN, TEXAS.

A MONTHLY JOURNAL OF MEDICINE AND SURGERY.

EDITED AND PUBLISHED BY

F. E. DANIEL, M. D.

ASSOCIATE EDITOR:

WITTEN BOOTH RUSS, M. D.,

San Antonio, Texas.

Published Monthly at Austin, Texas. Subscription price $1.00 a year in advance.

Eastern Representative: John Guy Monihan, St. Paul Building, 220 Broadway, New York City.

Official organ of the State Association of Health Officers, the West Texas Medical Association, the Houston District Medical Association, the Austin District Medical Society, the Brazos Valley Medical Association, the Galveston County Medical Society, and several others.

### THE PRESIDENT'S CASE.

The announcement on the seventh day after the injury and operation that the President was dying, and a few hours later that he was dead, came like a bolt from the blue. No one was more surprised than the medical men who had the case in charge. They all agree that the President died of gangrene,—but the attendants were not expecting gangrene, and do not know or say what kind of gangrene it was,—whether septic or trophic, or what caused it. All the leading medical weeklies of September 21st discuss the subject at length editorially, and it seems from a careful reading of those articles, that in the absence of *knowledge* of the cause of the gangrene, a "defective vitality on the part of the President" is "assumed."

No person who saw Mr. McKinley on his great tour, looking, as he did, the picture of health, able to speak every day and on all occasions, would for a moment suspect that he had not vitality enough to heal an incised wound in the abdomen! The chances for his recovery on the sixth day seemed all that could be desired.

I present the following extract from an editorial in *American Medicine,* written about that time:

"President McKinley's injury seems to be one offering exceptionally favorable prospects for recovery. The location of the wound in the stomach where peristaltic movement is comparatively limited and less likely to spread infection which would produce general peritonitis; the lack of injury to large vessels causing serious hemorrhage; the occurrence of the injury while in good health and while the stomach was nearly empty, and especially the immediate operation by thoroughly trained surgeons and the assurance of the best possible nursing and after treatment give every reason for hope of rapid recovery. Even under the unfavorable circumstances of the battlefield, Makin's recent "Surgical Experiences in South Africa" lead him to offer a comparatively favorable prognosis in cases of wounds of the stomach. With the best surgical skill and all the advantages of a well-equipped modern hospital the prospects should be still brighter."

Before *American Medicine* was mailed (September 14th, two days later), the President was dead.

I present the following from an exhaustive editorial in the *New York Medical News* of September 21st, a week after Mr. McKinley died:

"The usual causes of death after such injury and operation were escaped or removed or prevented, and their patient succumbed to a complication which is so rare that it could not reasonably have been anticipated and could not have been averted. The President died because he could not carry on the process of repair, and because the effort to do so was more than the vitality of the tissues involved could support. This, of course, excludes the possible presence of poison brought by the bullet or of destructive action by the pancreatic juices. If either of those was a factor, it needs only to substitute it in the statement for the assumed defective vitality of the patient. Whatever cause acted, it was unrecognizable at the operation and uncontrollable then or subsequently.

"There has been some criticism of the confident assurances of recovery made by those in attendance after the fifth day. To us the progress of the case up to that time appears fully to have justified those assurances and the public anxiety to have required them. The habitual causes of death had been escaped, and recovery could be prevented only by some rare complication which there was no reason to anticipate. The only irregular symptom was the frequency of the pulse, and that could be reasonably accounted for without invoking conditions that endangered life. There was not the slightest nausea, no complaint of discomfort, not the least abdominal pain; a soft abdomen in which percussion and firm pressure disclosed no sensitiveness; the bowels acting; the tongue clearing; the temperature falling; and a cheerful mind. Who can think that with such conditions on the sixth day the surgeons were not fully justified in believing that recovery was assured and, believing, in saying so? That a rare and at that time wholly unindicated complication should have then intervened was their and our misfortune. They did their work skillfully and judiciously, their behavior was dignified, restrained, and worthy of the best traditions of the profession, and they had the misfortune, when success seemed to have been secured, of seeing it overthrown by a complication which could not have been foreseen or avoided. They deserve our admiration and sympathy, not our criticism."

The *New York Medical News* thus makes several guesses and speaks of "unusual complications," but falls back on an "assumed defective vitality of the patient." All old surgeons of the civil war will recall how that soldiers, worn down by fatigue, loss of sleep, exposure to all kinds of bad weather, and often short rations, recovered from *amputations,* when the wound was pouring out pus by the pint ("laudable pus" they called it then; we knew nothing of sepsis or antisepsis), and healing by granulation or "second intention" was the rule. It seems remarkable that the medical press should seek to defend the medical attendant's want of skill or experience, their errors of omission and commission, their failure or error in diagnosis at the time of the operation; and their bungling management of the case and the autopsy, by such a flimsy pretext. He was not in the hands of the ablest men of the profession. With perhaps one exception, his attendants have no more than a local reputation, while there are men in America who are pre-eminent and are known all over the world for their skill, experience and success in abdominal surgery; others as famous as clinicians and pathologists. They were not called in consultation even. Such men, if present, would hardly have consented to a solid or semi-solid food (toast and eggs) on the fifth day, which, causing "indigestion," required, the attendants thought, castor oil and calomel! It is stated in the press disptaches that toast and eggs and castor oil and calomel were administered on the sixth day. I hope for the honor of the profession that it is not true. A trained nurse would have had more discretion than to so feed and dose a laparotomy case. One of the attending physicians says the pancreas was wounded. Three say it was not. One (known only as a yellow fever "expert") says the gangrene was caused by a poisoned bullet, but cannot name any poison which will cause gangrene. Another ridicules his suggestion and says the gangrene was produced by the pancreatic fluid, and so it goes.

The President did not have the benefit of the best medical and surgical skill in America, as he should have had. The full extent and nature of the injury was not ascertained at the operation, or there would be no dispute as to the injury inflicted. The ball was not found, but it was *assumed* to have lodged in the muscles of the back where, it was *assumed,* it would do no injury. Nor was it found at the autopsy! There was an X-ray machine in the nearby exposition building. It was not used.

In consideration of the foregoing and upon a careful analysis of the facts as given by the medical press, I am reluctantly forced to believe and say that the President did not have the best possible chance for his life; that the management of the case reflects no credit upon the medical profession, and that the death of the illustrious patient, under the circumstances, is a reflection on, if not a reproach to American surgery.

---

During the discussion of a paper on brain surgery before the Section on Surgery and Anatomy at the St. Paul meeting of the American Medical Association, Dr. R. H. M. Darbarn, of New York City, referred to a case occurring in his practice of complete left hemiplegia, which showed at the autopsy a blood clot covering a large portion of the left cerebral hemisphere. He stated that so far as he was able to learn only one other case of the kind had been reported, that of Dr. Joseph Bryant. If Dr. Darbarn will refer to the 1897 file of the *New York Medical Record* in one of the May numbers, he will find a paper by Dr. B. F. Kingsley, of San Antonio, Texas, containing an account of a case precisely similar. The patient in this case was a woman, 41 years old, who had been a sufferer for several years from a severe pelvic inflammation. Her paralysis, which developed during convalescence from an abdominal hysterectomy, was complete, both motor and sensory, and involved the right side. Accordingly the skull was opened on the left side, the operators being Drs. Kingsley and R. E. Moss. Absolutely no evidence of hemorrhage or other abnormal condition was discovered, and later a post mortem examination revealed a diffuse blood clot covering the greater part of the opposite hemisphere (the left), the side corresponding to the paralysis.

The logical explanation in this case, as in the cases of Dr. Darbarn and Bryant, is, of course, a failure of the motor fibres to decussate in the medulla. Such anomalies are extremely rare, and no well authenticated case should be overlooked. R.

